# Mechanisms of IgE‐mediated food allergy and the role of allergen‐specific B cells

**DOI:** 10.1002/1873-3468.70305

**Published:** 2026-02-19

**Authors:** Juan‐Felipe López, Özge Yilmaz Ardicli, Mübeccel Akdis

**Affiliations:** ^1^ Swiss Institute of Allergy and Asthma Research (SIAF) University of Zurich Davos Switzerland; ^2^ Division of Food Processing, Milk and Dairy Products Technology Program, Karacabey Vocational School Bursa Uludag University Turkey

**Keywords:** allergen‐specific immunotherapy, B cells, food allergy, IgG4, immune tolerance, oral immunotherapy

## Abstract

The substantial increase in food allergy prevalence during the last decades has made it a significant public health concern, affecting around 10% of the global population, especially children. Despite significant progress in understanding the general mechanisms of allergic sensitization, the development of oral tolerance remains a major challenge in advancing food allergy research and treatment. Additionally, each allergenic food source has a distinct immunological profile and tolerance trajectory, further complicating research efforts. Currently, oral allergen‐specific immunotherapy is the only treatment that can help build tolerance to certain food allergens over time—although treatment outcomes vary. While B cells have been described and studied for their pathogenic role in food allergy, recent evidence suggests that they also modulate allergic responses through various effector and humoral functions. Notably, despite their low frequency, recent knowledge on the molecular and functional characteristics of food allergen‐specific memory B cells has revealed important functions during both disease progression and therapeutic intervention. This review summarizes the current knowledge of IgE‐mediated food allergy, highlighting the role of B cells, especially allergen‐specific ones, in both disease and immune tolerance.

## Graphical abstract

Food allergy arises when allergen‐specific B cells preferentially produce immunoglobulin E (IgE) antibodies against harmless foods. This article explains the mechanisms driving IgE‐mediated reactions, highlights the central role of these B cells, and discusses how natural tolerance (NT) and oral immunotherapy (OIT) can reshape allergic immune responses. Created in biorender. Biorender, S. (2026) https://BioRender.com/he0e2xq.

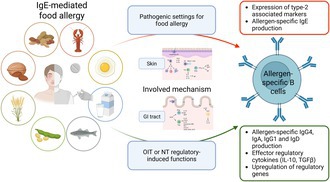

## Abbreviations

**AD**, Atopic dermatitis

**AIT**, Allergen‐specific immunotherapy

**APC**, Antigen‐presenting cells

**BAT**, Basophil activation test

**Bregs**, Regulatory B cells

**CSR**, Class switch recombination

**DCs**, Dendritic cells

**EPIT**, Epicutaneous immunotherapy

**GI**, Gastrointestinal

**Ig**, Immunoglobulin

**IL**, Interleukin

**ILC**, Innate lymphoid cells

**LC**, Langerhans cells

**MAT**, Mast cell activation test

**NT**, Natural tolerance

**OAS**, Oral allergy syndrome

**OFC**, Oral food challenge

**OIT**, Oral immunotherapy

**sIg**, Specific immunoglobulin

**SLIT**, Sublingual immunotherapy

**SPT**, Skin prick test

**Tfh**, T follicular helper

**Tregs**, Regulatory T cells

## Introduction

Allergy to food components is a major public health concern and represents a significant challenge in immunology and clinical practice, affecting approximately 7–10% [1, 2, 3] (adults: 10.8% and children ~ 8%) of the population, often leading to severe impacts on the quality of life [3, 4, 5]. It has been defined as an immunological reaction, mediated or not by the immunoglobulin E (IgE) molecule, after exposure to specific food antigens [6]. Clinically, these reactions can range from mild symptoms, such as discomfort or itching, to severe and potentially life‐threatening responses (anaphylaxis) [7]. Additionally, constant dietary exposure requires that the gastrointestinal (GI) tract serve as a tolerogenic organ, but the mechanisms behind this process are not fully understood [8]. Factors such as epithelial barrier integrity, gut flora composition, and immune system players influence whether food antigens trigger an allergic response or are tolerated [9].

The most common immune‐mediated mechanism driving the pathogenesis of food allergies is IgE‐mediated hypersensitivity [6]. Upon initial sensitization, allergen‐specific IgE antibodies are produced by B cells, which pass through a process of class switch recombination (CSR), regulated by cytokines such as interleukin (IL)‐4 and IL‐13 [10, 11]. These antibodies bind to high‐affinity FcεRI receptors on the surface of mast cells and basophils, and upon cross‐linking with a specific allergen, initiate cellular degranulation, resulting in the release of histamine and other proinflammatory mediators. This cascade results in the rapid onset of allergic symptoms [11]. However, B cells are not solely involved in promoting allergic reactions through antibody production; they can also play a role in tolerance induction through humoral and cytokine‐mediated effector mechanisms [12, 13]. Despite advances in understanding the mechanisms underlying food allergies, many aspects remain poorly understood, especially the role of allergen‐specific B cells in the pathogenesis and development of tolerance.

This review summarizes findings regarding the mechanisms associated with IgE‐mediated food allergy, with a particular focus on pathogenic and tolerogenic B‐cell responses to food allergens and the effect of oral immunotherapy and natural allergy outgrowth on B cells.

## General features of IgE‐mediated food allergy

Food allergy is a clinical disorder that can be broadly categorized into three groups: IgE‐mediated, non‐IgE‐mediated, and mixed (involving both mechanisms) [11]. These subtypes of the immune pathophysiology of food allergic responses have distinct symptom patterns and clinical manifestations [14].

IgE‐mediated food allergy is the most well‐characterized class and is associated with a risk of severe or even fatal reactions [11]. This type of food allergy can have different long‐term outcomes depending on the specific food involved. Cow's milk, egg, wheat, and soy allergy are relatively common in early childhood; however, a subset of affected children eventually outgrow these allergies as their immune systems mature. This phenomenon has been called natural tolerance (NT) [15]. Other allergen sources like peanuts, tree nuts, and shellfish tend to be persistent throughout the years, commonly continuing into adulthood [15]. Moreover, they are frequently correlated with more severe reactions, including anaphylaxis, making lifelong avoidance and careful management essential for affected individuals [11, 16].

Geographical location is also a determining factor in the prevalence of IgE‐mediated food allergies [16, 17]. Regional differences in climate, agriculture, and available food sources determine which foods are commonly consumed and introduced early in life. Furthermore, cultural dietary habits and food preparation practices play a significant role in sensitization patterns. These factors, combined with genetic and environmental influences, contribute to the varying rates and types of food allergies observed across different populations worldwide [11].

An interesting variant of IgE‐mediated food allergy is oral allergy syndrome (OAS), where individuals with allergen‐specific IgE (sIgE) antibodies against pollen allergens develop allergic responses to cross‐reacting proteins from fruits or vegetables. Symptoms typically include immediate oral itching, mucosal swelling, and sometimes abdominal pain. Since cross‐reactive IgE often targets heat‐labile and enzymatically configured epitopes, diagnostic testing for OAS should involve raw, unprocessed fruits or vegetables [18, 19]. Another form of IgE‐mediated food allergy requires sensitivity to the carbohydrate galactose‐α‐1,3‐galactose (alpha‐gal), found in red meat [20]. Unlike typical food allergies that target protein epitopes, this condition often develops following tick bites, which introduce alpha‐gal through the tick's saliva. Individuals may have tolerated red meat previously, before the onset of sensitization. Notably, reactions can be severe yet delayed, typically occurring 4–6 h or more after meat consumption [20, 21].

## IgE‐mediated food allergy diagnosis

Diagnosis still depends on refined versions of traditional tools (skin tests and serum IgE levels) alongside clinical history. It is important to take a detailed allergy history regarding time (symptoms usually develop within 30 min, sometimes up to 2 h) and triggers of the reaction [22, 23]. Some tools supported the clinical diagnostics as identifying IgE sensitizer through *in vivo* skin prick testing (SPT), *in vitro* measuring circulating sIgE or basophil activation test (BAT) if available [24]. Although SPT and sIgE levels are effective in detecting sensitization, their low specificity can result in overdiagnosis [23]. In contrast, the BAT demonstrates high specificity; however, results can be influenced by the allergenic source evaluated [25]. Therefore, oral food challenge (OFC) is still the gold standard tool for a definitive diagnosis of food allergy; but it is an approach with a higher risk for the patient [23, 26]. Clearly, both physicians and patients would prefer more accurate diagnostic tests to reduce the need for OFCs. While standard SPTs and sIgE tests are helpful, they have limitations and are not definitive on their own, which brings the necessity of a better understanding of the disease to improve diagnostic tools [27].

Although evidence has demonstrated the functional role of allergen‐sIgG, especially IgG4, as inhibitors of the allergic response, currently in food allergy, the heterogeneity of the results has not demonstrated a clear advantage of its measurements as a biomarker [28, 29]. Furthermore, multiplex platform *in vitro* assays could help to measure the sIgE directed toward a wide panel of food allergens, but as mentioned before, it is important to be aware of the impact on the diagnosis [30]. On the other hand, methods like IgE epitope‐based offer greater specificity but lower sensitivity compared to other IgE testing approaches, detecting antibodies that bind directly to allergen‐specific sites [31].

Promising novel *in vitro* diagnostic tests for food allergy include cellular assays that offer a key advantage as a functional assay, capturing the overall activity of IgE rather than just its presence or specificity, including the basophil activation test (BAT) and the mast cell activation test (MAT) [31, 32]. T‐cell profiling and evaluating activation in response to allergen stimulation may help distinguish clinical phenotypes in food allergy. For example, studies have shown that individuals with food allergies have a higher frequency of a specific cell type called T_H_2A compared to non‐allergic individuals, and these levels further increase following a positive double‐blind, placebo‐controlled food challenge [23, 33]. Currently, there are no methods based on B‐cell response or functionality. While some of these methods have shown promise in research settings, their integration into routine clinical diagnostics remains uncertain [23].

## Oral immunotherapy and current treatments

Food allergy immunotherapy is less commonly used in clinical practice compared to other types of AIT in allergic diseases. It has been classified into various types according to the route of administration, including oral immunotherapy (OIT), sublingual immunotherapy (SLIT), and epicutaneous immunotherapy (EPIT). Among these modalities, OIT is the most widely utilized approach [34, 35]. OIT typically involves the escalated regular ingestion of food allergens in milligram to gram quantities. The allergen is most commonly administered in flour form, mixed into a carrier food before consumption [36].

Although the range of OIT applications is quite broad, it also has drawbacks, as it can trigger severe allergic reactions. To prevent or mitigate this risk, approaches such as low‐dose OIT have been explored [34, 37]. While there is no clear consensus on the exact dosage adjustment, it generally involves a reduced maintenance and target dose, typically ranging from 5% to 40% of the dose used in conventional OIT. Notably, low‐dose OIT demonstrates comparable effectiveness to conventional OIT while offering an improved safety profile [34, 37, 38]. In combination with hypoallergenic foods, low‐dose OIT has the potential to promote tolerance with substantially fewer adverse events than conventional OIT. However, despite its improved safety profile, it may be less effective in achieving tolerance compared to conventional OIT [34].

Although achieving permanent tolerance is the ideal goal of the treatment, the absence of established biomarkers to reliably indicate tolerance, along with the often arbitrary nature of treatment discontinuation, has generally limited the use of the term “tolerance” [36]. Compared to other models of immunotherapy and allergic diseases, food allergies have higher relapse rates after treatment discontinuation [39]. Additionally, the nature of the allergen plays a role in the disease's natural history, with sustained tolerance being more commonly achieved, for example, in the case of cow's milk or egg than for peanuts or shrimp allergy [40, 41].

To date, there is very limited knowledge of the mechanism of sustained tolerance, especially in food allergy. However, it is important to remember that the immune system in the gut is highly complex, which also makes it vulnerable to dysregulation [8]. Hence, the difficulties in achieving food allergy tolerance are most likely due to the unique immunological system of the gut, the challenges of safely exposing allergic individuals to food proteins, and the lower efficiency of immunotherapy‐induced regulatory immune responses compared to venom and inhalant allergy treatments.

Mechanisms of allergen‐specific immunotherapy for food allergy are less known compared to inhalant allergy, and peanut allergy OIT is the most well studied [42]. A standardized oral immunotherapy product containing 300 mg of encapsulated whole peanut (AR101) has been approved and is administered daily, as demonstrated in the PALISADE study [43]. A significant reduction in allergen‐stimulated effector memory T_H_2A cells, defined as CRTH2^+^CD161^+^CD27^−^, was associated with a successful desensitization outcome, although the mechanism behind this decline remains unclear [33, 44]. Unlike earlier studies reporting a regulatory T‐cell (Tregs) response associated with food allergy [45], detailed flow cytometry in the PALISADE trial did not show a corresponding increase in peripheral Treg cells [46]. However, local gut induction of Tregs cannot be ruled out [47]. Individuals with peanut allergy after OIT also exhibit a decrease in basophil activation compared to the placebo group, which is probably mediated by the development of blocking antibodies [46]. Notably, oral immunotherapy basophil decreased activity is shown in the early stage, along with transient rises in peanut‐specific IgE, a substantial increase in the IgG4/IgE ratio [46], and reduced functional IgE‐binding capacity [45]. A recent breakthrough shows that early peanut oral immunotherapy in children under 4 years of age, especially with low sIgE and reduced basophil activation, may lead to sustained unresponsiveness for several months [48].

New treatments are emerging alongside traditional avoidance. Clinical trials have proposed EPIT as a promising therapeutic approach, particularly for peanut allergy in younger patients [49, 50]. EPIT involves applying a patch to the skin (the most advanced platform to date is called Viaskin) that contains a small amount of the allergenic protein. The goal is to expose the skin's immune cells to the allergen in a controlled manner, thereby promoting immune tolerance rather than triggering an allergic reaction [50]. Currently, there are no head‐to‐head studies comparing OIT and EPIT.

FDA‐approved options like Palforzia (peanut powder‐dnfp) and omalizumab (anti‐IgE). Other biologicals are under study, including dupilumab (anti‐IL4Rα), ligelizumab (anti‐IgE), and etokimab (anti‐IL33) [51]. Ongoing research aims to improve outcomes and long‐term treatment goals [52].

Advances in molecular allergology have led to the development of recombinant allergens, enabling more precise allergy diagnosis and better selection of candidates for allergen immunotherapy [42]. It is evident that, given the limited ability of current options to induce permanent tolerance, an ideal treatment remains elusive. Nevertheless, emerging therapies offer promising prospects for individuals with food allergies.

## Epithelial barrier in allergic sensitization to food allergens

The role of skin and mucosal epithelial barriers is critical for defending the body against environmental agents by functioning as physical, chemical, and immunological shields. Regarding pathogens and allergens, they form the first line of defense, avoiding infiltration, helping to preserve structural and functional integrity, and maintaining overall homeostasis [53, 54]. The physical thickness and strength of the skin barrier is given by a robust, stratified, and multicellular layer system. The skin‐gut axis plays a key role in the development of IgE‐mediated food allergy [55]. Skin barrier dysfunction has been correlated with food allergy, explained by the concept of transcutaneous allergen sensitization, particularly in patients with atopic dermatitis (AD) [56, 57]. Additionally, impairment of tight junctions in the epidermal granular layer constitutes a factor that also contributes to barrier dysfunction and immune dysregulation in AD [56, 58, 59]. Human mutations in genes involved in skin barrier integrity, such as *FLG* and *SPINK5*, highlight the critical role of a healthy epithelial barrier in the independent risk of developing food allergy to peanut [60, 61, 62]. Beneath the epidermal layer, the dermis contains different immune cells that could be actively involved in the sensitization process in food allergy, including innate cells like mast cells, Langerhans cells (LCs), and innate lymphoid cells (ILCs), as well as adaptive immune system cells like resident lymphocytes [55].

Additionally, the EPIT therapeutic approach highlights the skin as a target organ for inducing immune tolerance. It operates through a distinct skin‐driven pathway in which allergens are captured by LCs and dermal dendritic cells that migrate to skin‐draining lymph nodes to promote Tregs response [50, 63]. EPIT appears to gradually induce long‐lasting, multifunctional Tregs with broad tissue‐homing potential and stable epigenetic reprogramming (such as sustained *Foxp3* demethylation and *Gata3* silencing) [64]. Mouse models identify LCs as key mediators of tolerance via PD‐L2– and TGF‐β–dependent mechanisms [63, 65], while clinical data demonstrate modulation of allergen‐specific Th2 and IL‐10^+^ T cells during EPIT [66]. As a result of industrialization and westernization, the amount of exposure to air pollution, harmful chemicals, processed foods, and additives has contributed to microbiome imbalance, an increase in inflammatory conditions, and the development of type 2‐biased immune responses [56, 67]. A better understanding of these processes will eventually help to develop new preventive and therapeutic strategies. The cellular and humoral immune‐related mechanisms of food allergy will be explored in greater detail in the following sections.

## Immune players in IgE‐mediated food allergy and tolerance to food antigens

The reactions in IgE‐mediated food allergy are characterized by a rapid onset after exposure to the trigger food, which can be from minutes to a few hours [14]. Subsequent exposure to food allergens induces IgE‐mediated activation of immune effector cells, including mast cells and basophils, leading to the rapid release of histamine and other inflammatory mediators [68]. This leads to immediate allergic symptoms, followed by sustained inflammation driven by type 2 cytokine responses, particularly IL‐4, IL‐5, and IL‐13.

Tuft cells, also known as brush cells, are specialized epithelial barrier cells found in the intestine and trachea that produce IL‐25 (also known as IL‐17E), a member of the IL‐17 cytokine family, in response to inflammation [55, 69, 70]. It has been suggested that tuft cell activation by microbial metabolites, such as succinate, may lower the threshold for food allergic responses, enhancing epithelial permeability and mast cell activation [71].

Activation of type 2 innate lymphoid cells (ILC2s) by epithelial‐derived cytokines, such as IL‐33 and thymic stromal lymphopoietin (TSLP), has been shown to play a key role in the development of food allergy [55]. ILC2s have been demonstrated to be an early source of IL‐13 during peanut exposure, which promotes the development of T follicular helper (Tfh) cells and germinal center B cells, leading to the production of allergen‐specific IgE antibodies in mice [72]. Furthermore, the expansion of intestinal mast cells is linked to a higher susceptibility to food‐induced anaphylaxis, and increased intestinal mast cell levels are correlated with greater severity of anaphylactic reactions [55].

CD4+ cells, especially Tfh, play a fundamental role in the sequential class switching of memory B cells in germinal centers to convert them into IgE‐producing cells. These cells have been called Tfh13, which produce IL‐13, IL‐4, and IL‐5 and express the transcription factors GATA3 and BCL6. Peanut‐allergic patients have shown increased frequencies of this specific cell phenotype [73].

**Fig. 1:**
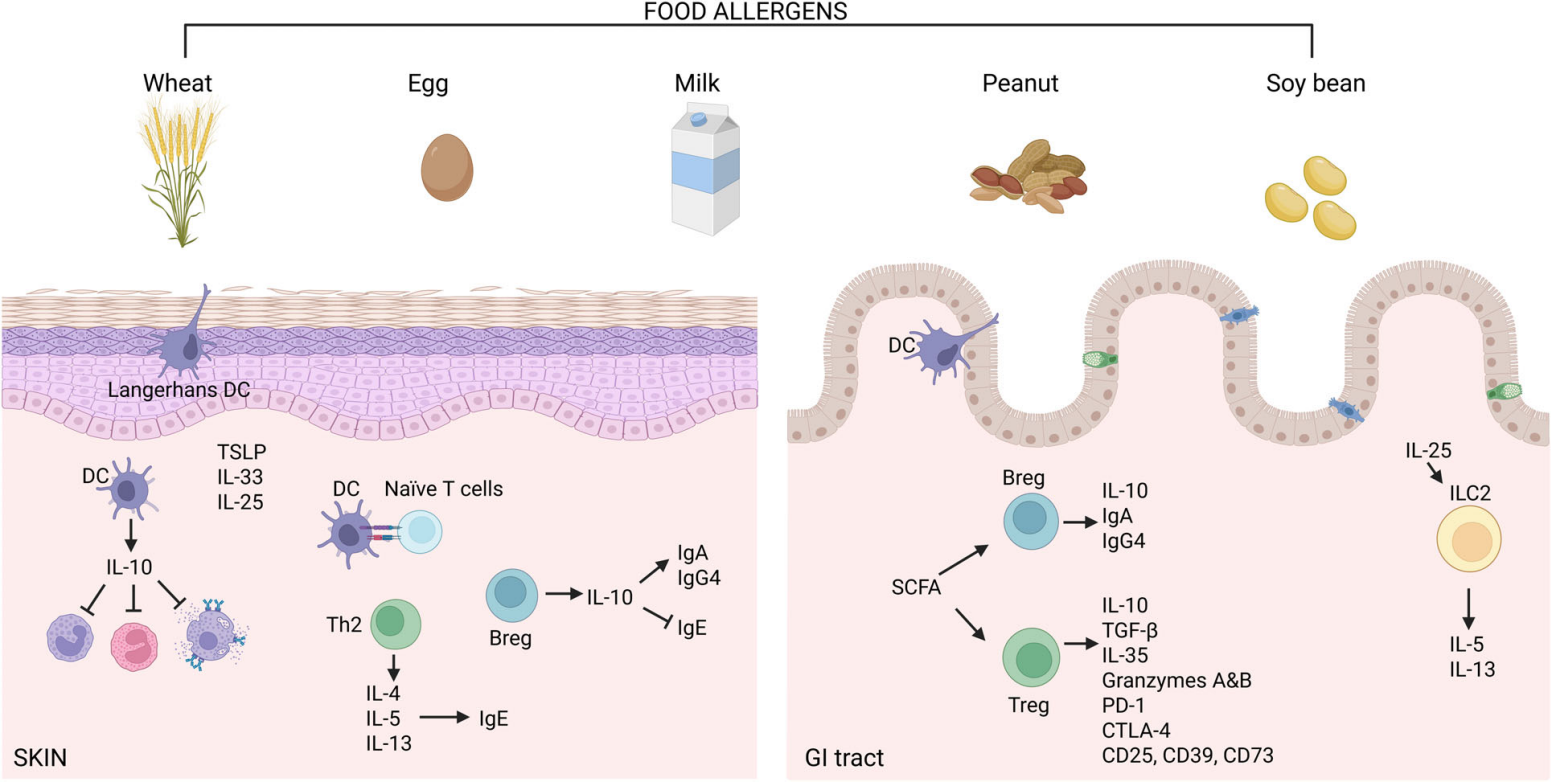
Different mechanisms of food allergy in skin and gastrointestinal tract. The distinct immunological pathways triggered by food allergens such as wheat, egg, milk, peanut, and soybean in the skin (left) and the gastrointestinal (GI) tract (right). In the skin, allergens activate Langerhans cells and dermal dendritic cells (DCs), promoting the release of epithelial cytokines (TSLP, IL‐33, IL‐25) that favor Th2 polarization. Th2 cells secrete IL‐4, IL‐5, and IL‐13, driving IgE production and allergic sensitization. Regulatory B cells (Bregs) can counteract this by releasing IL‐10, which supports class switching to IgA and IgG4 while suppressing IgE. In the GI tract, dietary antigens are taken by intestinal DCs, which contribute to the induction of Bregs and regulatory T cells (Tregs), especially in the presence of microbiota‐derived short‐chain fatty acids (SCFAs). Bregs produce IL‐10, IgA, and IgG4, while Tregs mediate tolerance through IL‐10, TGF‐β, IL‐35, granzymes A and B, and inhibitory receptors (PD‐1, CTLA‐4) and markers (CD25, CD39, CD73). IL‐25 can also activate ILC2s, which promote Th2 cytokine production (IL‐5, IL‐13). Created in biorender. Biorender, S. (2026) https://BioRender.com/7czxmua.

Overall, the tolerance process to food antigens is a result of multiple coordinated immune mechanisms, including suppression of type 2 immune response activity, induction of Tregs, reduced IgE production by B cells, increased production of IgA and IgG4 antibodies, and inhibition of effector T‐cell migration to tissues (Fig. 1). Additionally, it involves the activation of IL‐10‐producing dendritic cells (DCs) and the suppression of basophils, eosinophils, and mast cells [56]. In the mucosa, a significant amount of Treg cells is necessary to balance the tolerance to daily oral antigen consumption. Antigen‐presenting cells (APCs) that primarily display food‐derived antigens are predominantly found within the cDC1 subset of DCs. These cells possess an inherent ability to encourage the development of Tregs, crucial for maintaining immune tolerance [74]. Expansion of Treg cells is a result of a balanced gut microenvironment where commensal microbiota produce retinoic acid and metabolites, such as short‐chain fatty acids, that are fundamental for the maintenance of this cell population in the gut [56, 75]. Treg cells provide the production of suppressor cytokines including IL‐10, IL‐35, and TGF‐β, but also regulated through effector mechanisms like cell cytolysis by granzymes A and B, direct targeting of DCs through inhibitory programmed cell death 1 (PD‐1) and cytotoxic T lymphocyte‐associated protein 4 (CTLA‐4) cell surface molecules, and metabolic disruption of effector cells (CD25, CD39, CD73, cAMP, and adenosine) [56, 76]. It is worth noting that forkhead box P3 (FoxP3)^+^ Treg cells are essential for the establishment of oral tolerance [56, 77]. Allergen‐specific response is essential for both disease and tolerance development. It has been shown that tolerant individuals showed significantly higher frequencies of allergen‐specific Treg cells (16.85 vs 4.91%) compared to allergic children. Functionally, depleting the CD25^high^ subset before culture led to enhanced effector T‐cell expansion, suggesting that these Tregs actively suppress allergic T‐cell responses [78].

B cells play a crucial role in developing and regulating food allergies, particularly those mediated by IgE antibodies. Chronic allergen exposure promotes the accumulation of long‐lived IgE plasma cells in the bone marrow, thereby sustaining enduring serological memory and contributing to the persistence of allergic disease [79]. Regulatory B cells (Bregs) can also contribute to immune tolerance primarily through the production of IL‐10 and IgG4, as demonstrated in experimental models of infection, allergic inflammation, and immune tolerance [56, 80, 81]. A deeper understanding of the mechanisms underlying pathological and tolerogenic players is essential for advancing new preventive and therapeutic strategies.

## B cells in IgE‐mediated food allergy and tolerance

**Fig. 2:**
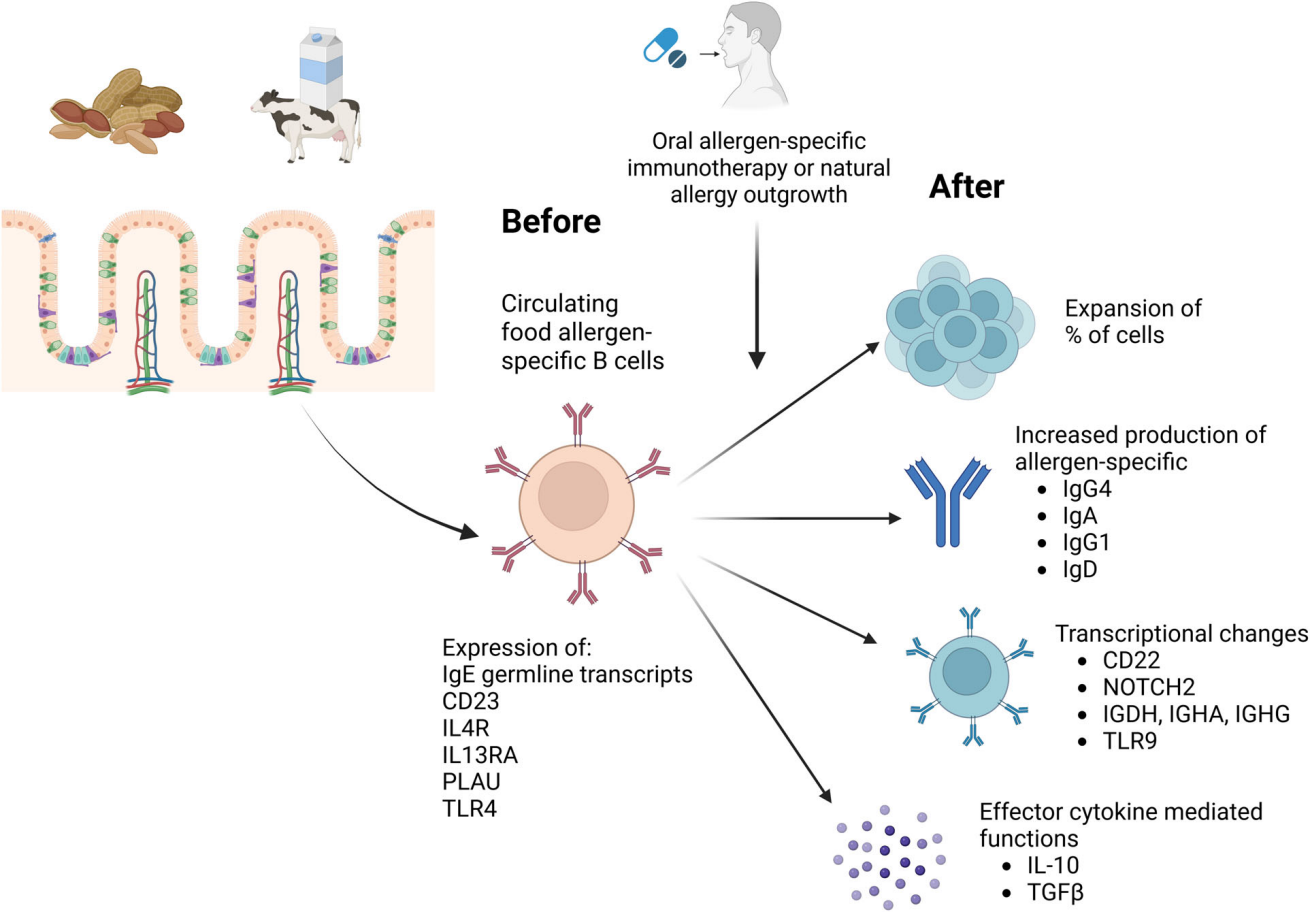
Changes induced in allergen‐specific B cells after allergen‐specific immunotherapy and natural allergy outgrowth. Few studies have described features related to allergen‐specific B cell in allergic individuals, mainly the association with the expression of IgE germline transcripts and the expression of CD23 and IL4R. After allergen‐specific immunotherapy or in natural allergy outgrowth, cells experience changes in their frequencies, transcriptional profile, and effector functions. Created in biorender. Biorender, S. (2026) https://BioRender.com/los99xq.

Allergic reactions to food can be mediated by IgE antibodies produced by allergen‐specific B cells [24]. However, B cells also act as modulators through the production of different allergen‐specific antibody isotypes, and effector regulatory mechanisms may be induced following persistent food consumption [10, 12, 82]. Additionally, findings in natural food allergy outgrowth have shown that B cells present specific changes that can be involved in this outcome [13, 82]. In contrast with IgE‐mediated food allergy, in non‐IgE‐mediated diseases like eosinophilic esophagitis, IgG4 has been proposed to have a possible pathogenic role as it presents elevated levels in patients [83]. Understanding the mechanism of B‐cell response in food allergy can help to gain insights into the diagnosis, prognosis, and therapeutics of this condition (Fig. 2).

## Humoral response to food allergens

The sensitization process in food allergy mainly occurs through allergen exposure in the epithelial GI [84] but also through the skin as mentioned in the previous section. The IgE antibody response in food allergy is still not fully understood; however, specific features have been described. In peanut‐allergic patients, a higher abundance of IgE‐expressing B cells was found in the GI and peripheral blood. It seems that IgE class switch recombination (CSR), plasma cell differentiation, and allergen‐specific IgE production occur locally in the GI [85]. Interestingly, IgE clones are shared by different peanut‐allergic individuals. It has also been demonstrated that most IgE‐producing cells result from sequential and not direct CSR, mainly from IgA or IgG isotype memory B cells. This sequential class switching is also associated with higher affinity antibodies [85, 86, 87, 88]. A mouse model of peanut allergy also demonstrated that the timing of allergen exposure is required for IgE generation, and that Tfh cells expressing Fgl2 can repress IgE induction [89].

In addition to their response producing allergen‐specific IgE antibodies, B cells can also generate other isotypes, which have been hypothesized could protect against food allergies. In a mouse model of food allergy, allergen‐specific IgG through FcγRIIB restores and sustains the tolerance to food antigens by a mechanism that involves mast cells and Treg cells [90, 91]. However, among IgG isotypes, IgG4 has been closely linked to the inhibition of allergen activity. Among the characteristics that make IgG4 a blocking/protective isotype in IgE‐mediated responses are its inability to activate complement pathways and a process called Fab arm exchange, which endows it with both bi‐specificity and monovalency [92]. While the exact role of allergen‐specific IgG4 in food allergy is not fully understood, several studies suggest that it may be associated with the development of tolerance rather than allergic reactions, particularly when considering IgE levels (IgG4/IgE ratio) [23]. Consumption of major food‐allergenic sources like egg, milk, and peanuts in early life increases the levels of specific IgG4, and in the case of peanuts, it has been associated with a decreased risk of allergy development [93]. Additionally, food‐sIgG4 has also been correlated with the natural food allergy outgrowth outcome or prediction of tolerance [94, 95, 96]. Functionally, depletion of IgG4 in sensitized but tolerant children to peanuts and after OIT restored the basophils and mast cells activation, indicating the important role of IgG4 in the prevention of IgE‐mediated reactions [28].

Clinical and experimental evidence has tried to resolve whether IgA could be important for the development of food allergies. Associations between IgA deficiency and food allergy incidence have been established [97]. Increased serum and salivary sIgA concentrations have been reported in egg allergy NT and peanut OIT, respectively [98, 99]. Conversely, gut sIgA evaluated in a cohort of peanut and egg allergy could not predict allergy development or tolerance and sIgA and sIgE differ in epitope specificities [100]. Hence, research to understand the real impact of sIgA on food allergy needs to be done.

Recently, secreted IgD has been reported to have a bystander effect in type 2 responses. IgD can act by inhibiting IgE degranulation but also inducing the production of type‐2 cytokines in basophil and mast cells [101, 102, 103]. Regarding food allergy, milk and egg OIT increases the allergen‐specific IgD response and sIgD to egg components was also associated with a lower risk of anaphylaxis reactions [101, 104, 105]. Evidence of sIgD response is still scarce to conclude the effect of this antibody isotype in the protection or enhancement of the IgE‐mediated food allergy.

## B‐cell effector mechanism of tolerance

Since dietary antigens are constantly in contact with our immune system and epithelial cells, tolerance mechanisms are crucial for avoiding hypersensitivity reactions to them. Findings have suggested that the tolerance mechanism mediated by B cells could be impaired in food allergy. In an experimental murine model of food allergy to cow's milk‐derived casein, an IL‐10‐producing Bregs phenotypically CD5+ subset was found in mesenteric lymph nodes, which interact with Treg cells Foxp3+ and are capable of suppressing IgE‐mediated anaphylaxis to the allergen [106]. Since mice are unable to produce IgG4, the role of Bregs observed in mouse models may be of limited relevance when translating these findings to humans, where IgG4 plays a significant role in immune tolerance. This raises important considerations when interpreting data from mouse allergy models, as key mechanisms underpinning tolerance in humans (such as IgG4‐mediated immune regulation) may not be adequately reflected in these models.

Supporting this role of Bregs, in human subjects with milk allergy, IL‐10‐producing Bregs were decreased compared to a milk‐tolerant group after casein stimulation [107]. In addition, a proinflammatory status mediated by circulating B cells producing IL‐8 was associated with persistent egg allergy [108]. All these findings support the role of effector B cells in the tolerance maintenance, mainly to food antigens. However, there are still some gaps in terms of antigen specificity and direct or indirect mechanism.

## Allergen‐specific B cells and effects of allergen‐specific immunotherapy in B‐cell response in food allergy

As was mentioned before, the IgE‐CSR is more likely to be sequential from allergen‐specific B cell precursors of the memory compartment. Recently, two independent studies found CD23 and IL‐4Rα as markers associated with IgE germline transcripts and defined this population as precursors of IgE‐producing cells [109, 110]. In peanut‐allergic individuals, CD23+ memory B cells were higher in patients with higher titers of peanut‐sIgE [109]. From the entire repertoire of circulating memory B cells, allergen‐specific ones are scarce, representing a tiny percentage of them, depending on the antigen exposure model (0.03–0.3%) [12, 111, 112, 113]. Hence, data regarding allergen‐specific cells is limited due to the difficulties in the detection methods and the limited amount of biological material to perform multi‐omics analysis. Few studies have been performed on peanut and milk food allergy models. Interestingly, increased frequencies of circulating Ara h 1, Ara h2, and Bos d 9 allergen‐specific B cells have been reported after peanut and milk OIT; however, this is not related to a pathogenic effect [13, 111, 112]. Single cell approaches in B cells from allergic peanut individuals allowed determining that there is convergence in antibody response from allergen‐specific cells of different donors, which is remarkable due to the low chances of similarity [114]. Newly, the effects of cow's milk OIT on allergen‐specific B‐cell responses have been elucidated. It has been shown that after OIT and in natural allergy outgrowth, Bos d 9‐specific B cells changed their transcriptional profile. Interestingly, these changes are associated with specific clinical outcomes like desensitization, remission, or even natural allergy overgrowth. Among the candidates that can help understand the mechanisms underlying the sustained tolerance response or its failure are B‐cell tolerance and marginal zone development markers like CD22, TLR9, NOTCH2, and IGHD [13]. Besides the expansion and transcriptional changes, these cells are functionally different in humoral and effector response, producing significantly more allergen‐sIgG4 and regulatory cytokines like IL‐10 and TGF‐β [13]. There are still open questions regarding changes at the single‐cell level and if the effect of immunotherapy is on preexisting or newly induced cells.

## Conclusions and perspectives

The intricate mechanisms of food allergy and the dual role of allergen‐specific B cells highlight both the complexity of the immune response and the potential for targeted therapeutic interventions (Table 1). While significant progress has been made in understanding the pathogenesis of food allergies, several key areas warrant further investigation. The plasticity of B cells, particularly their ability to switch between pro‐allergic and regulatory phenotypes, presents an intriguing avenue for future research. Harnessing this plasticity could lead to novel immunotherapies that promote tolerance induction. Additionally, the identification of specific B cell subsets and their associated biomarkers may enable more precise diagnostic tools and personalized treatment approaches. As our understanding of the microbiome's influence on immune responses deepens, exploring the interplay between gut bacteria and allergen‐specific B cells could unveil new strategies for allergy prevention and management. Furthermore, advances in single‐cell technologies and multi‐omics approaches promise to provide unprecedented insights into the heterogeneity of B‐cell responses in food allergy.

**Table 1:** Key take‐home messages on the role of B cells in food allergy and immune tolerance. This table summarizes current insights into B‐cell–mediated mechanisms in allergy development and resolution.

Take‐home messages
B cells are not only key players in IgE production and allergy development but also essential modulators of immune tolerance via IL‐10, IgG4, IgA, and possibly IgD
Allergen‐specific IgE‐producing B cells and class switch recombination (CSR) occur predominantly in the GI mucosa, indicating site‐specific immune responses
High food‐specific IgG4 levels correlate with natural resolution of allergy and successful immunotherapy, acting as functional blocking antibodies. However, it has not been defined yet as a biomarker
Oral immunotherapy (OIT) induces transcriptional and functional shifts in allergen‐specific B cells, increasing IL‐10 and IgG4 production, even in natural outgrowth cases

## Conflict of interest

MA received research grants from the Swiss National Science Foundation. She is a Scientific Advisory Board member of Stanford University‐Sean Parker Asthma Allergy Center, CA; an Advisory Board member of the LEO Foundation Skin Immunology Research Center, Copenhagen; and a Scientific Co‐Chair of the World Allergy Congress (WAC) Istanbul, 2022, Scientific Programme Committee Chair, EAACI. J‐FL and OYA do not declare any conflict of interest.

## Author contributions

JFL, OYA, and MA contributed equally to this manuscript.

## Funding

This study was funded by the Swiss National Science Foundation (SNSF/SNF) Grant No. 310030‐201053/320030‐159870/310030_201053/1(PI: Mübeccel Akdis).
